# Geographic and Demographic Disparities in Late-stage Breast and Colorectal Cancer Diagnoses Across the US

**DOI:** 10.3934/publichealth.2015.3.583

**Published:** 2015-08-28

**Authors:** Lee R Mobley, Tzy-Mey (May) Kuo

**Affiliations:** 1 Spatial Science and Health Economics, School of Public Health and Andrew Young School of Policy Studies, Georgia State University, Atlanta, GA, USA; 2 Lineberger Cancer Center, University of North Carolina at Chapel Hill, Chapel Hill, NC, USA

**Keywords:** translational mapping, multilevel modeling, cancer control, late-stage cancer diagnosis, geographic disparity, demographic disparity

## Abstract

**Problem:**

In 2009, breast cancer was the most common cancer in women, and colorectal cancer was the third most common cancer in both men and women. Currently, the majority of colorectal and almost 1/3 of breast cancers are diagnosed at an advanced stage in the US, which results in higher morbidity and mortality than would obtain with earlier detection. The incidence of late-stage cancer diagnoses varies considerably across the US, and few analyses have examined the entire US.

**Purpose:**

Using the newly available US Cancer Statistics database representing 98% of the US population, we perform multilevel analysis of the incidence of late-stage cancer diagnoses and translate the findings via bivariate mapping, answering questions related to both *Why* and *Where* demographic and geographic disparities in these diagnoses are observed.

**Methods:**

To answer questions related to *Why* disparities are observed, we utilize a three-level, random-intercepts model including person-, local area-, and region- specific levels of influence. To answer questions related to *Where* disparities are observed, we generate county level robust predictions of late-stage cancer diagnosis rates and map them, contrasting counties ranked in the upper and lower quantiles of all county predicted rates. Bivariate maps are used to spatially translate the geographic variation among US counties in the distribution of both BC and CRC late-stage diagnoses.

**Conclusions:**

Empirical modeling results show demographic disparities, while the spatial translation of empirical results shows geographic disparities that may be quite useful for state cancer control planning. Late stage BC and CRC diagnosis rates are not spatially random, manifesting as place-specific patterns that compare counties in individual states to counties across all states. Providing a relative comparison that enables assessment of how results in one state compare with others, this paper is to be disseminated to all state cancer control and central cancer registry program officials.

## 1. Introduction

Several studies have examined the predictors of stage at diagnosis for breast and colorectal cancers. Using Surveillance, Epidemiology, and End Results (SEER)-Medicare data, studies have examined low SES, marital status, race or ethnicity, distance to closest provider, managed care penetration, area screening rates, or residence in a racially segregated community as predictors of late-stage diagnosis [1],[2]. Other studies which examined whole cancer populations in multiple states or regions in the US found that factors such as area poverty or deprivation, lack of personal insurance, or percent uninsured in the area have associations with late-stage BC diagnosis [3],[4]. Another study found that patients privately insured or insured by Medicare plus supplemental plans had lower likelihood of being diagnosed at advanced stages of cancer than persons with other types of insurance, with highest rates among uninsured and Medicaid insureds [5].

A recent paper examined predictors of late-stage BC and CRC diagnoses during 2001-2005 in 11 SEER Registry states [1]. The analysis was representative of persons aged 65 or older who were eligible for Medicare, and linkages with the Medicare beneficiary files allowed ascertainment of marriage status and socioeconomic status (via the dual eligibility indicator). The paper examined how the state-level variation in health insurance regulatory environments predicted late-stage cancer diagnosis outcomes through cross-level interactions with community-level factors that were specified from the perspective of the cancer patients in those communities.

Health insurance regulatory environments vary considerably across the United States, and several studies have examined the impacts of health insurance regulation and mandates on insurance premiums and rates of uninsured individuals [6]–[9]. One study concluded that opening up competition among plans across states would lower premiums and increase value in the benefits packages offered to consumers [10]. The late-stage cancer predictors paper [1] was novel, in that no previously published studies concerning health insurance regulations or mandates had examined their impacts on health outcomes, and conversely no previous studies of population cancer outcomes had included state-level factors such as health insurance regulations.

**Figure 1.**
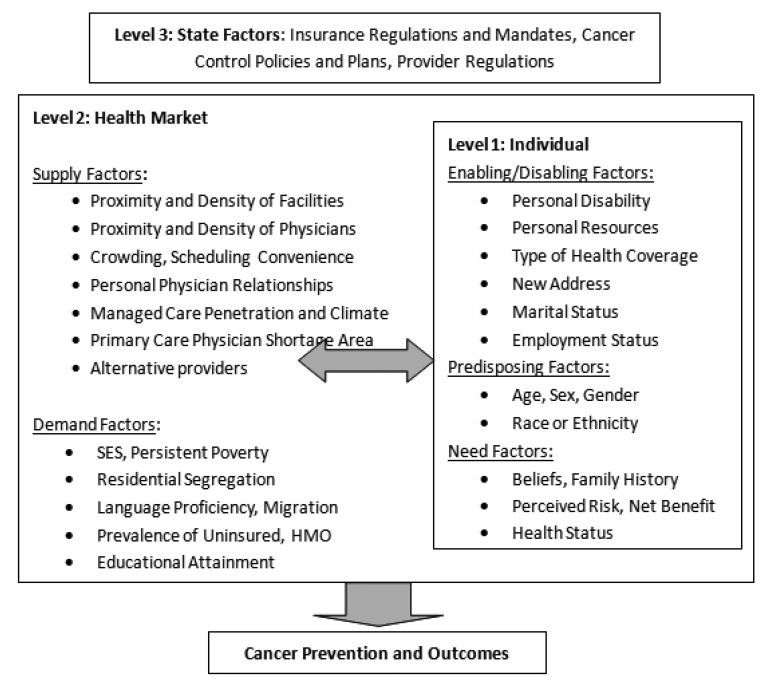
Spatial Interaction in Three Levels of Influence Associated With Late-Stage Cancer Diagnosis Across the Heterogeneous United States.

Using traditional multilevel modeling [11] with a random intercepts specification to produce robust area-level predictions from the large person-level analytic file, the researchers [1] pooled the data across the 11 SEER registry states. They used a very similar conceptual model to the one used in this paper (Figure 1, methods section below), to guide hypotheses regarding effects of particular insurance regulations or mandates that might impact cancer outcomes, limiting attention to those regulations or mandates that actually varied across the 11 states studied. The authors found that several state-level insurance regulation variables were important predictors of late-stage breast and colorectal cancer diagnoses in the 11 states. Cross-level interactions between state and health market level factors were also significant. States that mandated insurers to cover inpatient hospitalization stays after mastectomy had different outcomes than states with no such mandate. In the three states with the largest proportion of minority populations (California, Georgia, and New Mexico) this mandate was associated with significantly reduced probability of late-stage BC in more residentially segregated communities. People were matched by race or ethnicity to residential segregation measures, creating a single race-matched index variable that was assumed to be directly associated with greater social support for healthy behaviors. The reductions in probability of late stage breast cancer diagnosis from the three policy models were -7%, -3%, and -7%, respectively (these are net effects from level and interaction terms considered together, evaluated at the state mean of the race-matched residential segregation index).

In modeling late-stage CRC diagnosis, the authors focused on state insurance ‘continuity of care’ mandates (i.e., that insurers must allow patients to keep established providers when they become seriously ill or changed insurance plans). They found that states which mandated continuity of care had significantly lower rates of late-stage CRC in counties with the highest proportion of poor or chronically ill cancer patients (measured by a SES variable available for the Medicare enrollees studied). They found that, when states *do have* continuity of care laws, the effect of living in lower SES counties was, on average, a modest reduction in the state's rate of late stage diagnosis. However, when there were no continuity of care laws, the effect of living in lower SES counties was, on average, a much higher rate late-stage diagnoses. That paper [1], examining cancer registry populations in 11 SEER Registry states, provided significant albeit preliminary evidence that the state insurance regulatory environment does impact whether people are diagnosed with breast or colorectal cancer at later stage.

The unique contribution of the current paper is to build on and update the past literature and broaden its geographic scope, by demonstrating methods by which the newly available, combined state cancer registry datasets might be used to translate information regarding areas where problems in cancer control are more prevalent. The study is unique in that it examines these recently available population data from cancer registries across the US, including all newly diagnosed breast and colorectal cancers, for patients of all ages, over the six-year period 2004–2009. The US exhibits considerable heterogeneity across states in many aspects including health insurance regulatory environments, sociodemographic and socioecological factors, environmental factors, health market dynamics and disease burdens. These factors together explain observed incidence of late-stage diagnoses, and can be used to robustly predict places where cancer control problems associated with one or both of these cancers are more prevalent. Such predictions are more robust than mapping of raw rates of late-stage diagnosis, as they are adjusted for uncertainties associated with small sample sizes which make raw rates less reliable indicators of problem areas.

## 2. Methods

### 2.1. Conceptual Model

We adapt the conceptual model from the study described above [1] which posits three levels of influence on cancer control outcomes: person, health market, and state. Figure 1 (below) summarizes a wide spectrum of various influences across these three levels. At the state level, state insurance regulations are among other macro-level factors that can influence outcomes. Among the state, health market and individual level factors, more aspects are listed than can be modeled using currently available data or computing power. The particular measures used in modeling, at each level of influence, are described in Table 1 and in section 2.3 below.

Few papers to date in the health outcomes literature have explicitly considered what the relevant levels of influence may be, or why this would matter in empirical models. One paper examined this explicitly, and posited different interpretations for the same variables at different levels of aggregation [12]. Our conceptual model is based on that study, and includes a blend of several models used by researchers that evolved over time. The foundation is the behavioral model of utilization developed by Aday and Andersen [13], which defines the predisposing, enabling, and need characteristics of individuals and several factors associated with health care access, but does not include other important contextual factors [14],[15]. Subsequent models of health utilization or outcomes commonly used contextual factors at multiple levels within a socioecological framework [16]–[19]. Explicit consideration of the relevant ‘zone of influence’ for ecological variables has been a more recent topic in the health market or neighborhood definition literature [12],[16]–[24].

### 2.2. Study Population and Other data

We examined cancer cases diagnosed during 2004–2009 from the United States Cancer Statistics (USCS) database, which is a population-based surveillance system of cancer registries with data representing 98% of the U.S. population. The database was developed by a joint effort of the Centers for Disease Control and Prevention (CDC) and the National Cancer Institute (NCI) to provide a single, pooled-state database of reconciled, comparable cancer information geocoded at the local level to facilitate cancer control planning and evaluation [26]. Included in the database is information on demographics (age, gender, race, ethnicity), tumor characteristics, and geographic location (state, county) at diagnosis. This comprehensive database is now available inside National Centers for Health Statistics (NCHS) and Census Research Data Centers (RDCs) to qualified researchers [27]. We restrict the sample to all persons having a first breast or colorectal cancer diagnosis during 2004–2009. We excluded records when BC or CRC were not the primary cancers, records with unknown cancer stage or unstaged cancer, or when diagnosis was by autopsy or death certificate (< 1% of all cases). For BC we excluded males. These restrictions yielded a CRC study population of 558,568 individuals and a BC study population of 981,457 individuals.

Most states participate in the USCS registry data system, but four states do not allow use of county of residence information (Illinois, Michigan, Missouri, Ohio). Three states did not participate at all over the timeframe of this study (Kansas, Maryland, Minnesota). We excluded these 7 states and an additional state, Virginia, because data were not available until 2007. We also excluded Hawaii and Alaska because of missing contextual data (Alaska) and the fact that each island is a county (Hawaii) which results in identification of specific counties, which is not permitted by the NPCR. These exclusions result in the 40 remaining states being included in the analysis.

Other Data Sources: County level data describing contextual characteristics of communities obtained from the RTI Spatial Impact Factor Database [28], which derives from numerous sources. State-level data describing insurance regulatory environments were downloaded from the National Conference of State Legislatures databases [29]. Data description, sources, and brief rationale for inclusion of each covariate are provided in Table 1. Sample statistics are also provided in Table 1, all based on the individual level of observation.

Figure 1 depicts the blended health behavioral and socioecological perspectives in a hybrid spatial interactions model based on the model of health care access and utilization first developed by Khan and Bhardwaj [25] for the World Health Organization. Utilization of healthcare is described as a process involving interaction between the characteristics of the health care service system, potential users, and the social and physical features of places. To this spatial interaction model we have added the regulatory realm of influence because it does vary so much across the US. The spatial interaction among all of these various constructs and levels of influence are illustrated in Figure 1.

**Table 1.** Multilevel Model Variables: Description, Rationale, Source, and Sample Statistics.

			BC		CRC	
**Outcome**: whether cancer patient was diagnosed at a late stage (regional or distant = 1, else = 0)	Late stage diagnosis is indicative of lack of knowledge regarding personal cancer risk, or the importance or availability of screening; lack of timely or proximate access to services, lack of funds to pay for, and cultural or other barriers related to utilization of timely cancer screening.	SEER and NPCR cancer registry data made available through NCHS	mean	sdev	mean	sdev
			0.308	0.461	0.543	0.498
**Person-level predictors**		Research Data Centers, covering 2004–2009: http://www.cdc.gov/rdc				
female (binary)	Only females are included in BC study. Although males do have BC incidence, the numbers are few. Both male and female are included in the CRC study, with male designated as the reference group.		1.000	0.000	0.487	0.500
black (binary)	The national statistics cite blacks as a disadvantaged group, with worse outcomes relative to whites, the reference group.	BC N = 981,457CRC N = 558,568	0.101	0.301	0.112	0.315
race all other (binary)	All other races and ethnicities were combined to make the model more parsimonious, relative to whites, the reference group. Includes 8% Hispanic, 3% Asian, 0.5% Native American, 0.8% other.		0.126	0.332	0.124	0.329
age < 49 (binary)	BC screening protocols recommend to start screening at age 40 for higher risk women		0.226	0.223	0.111	0.275
age 50–64	CRC and BC screening protocols recommend to start screening at age 50 for average risk individuals; this is the prime age bracket for both screening modalities.		0.366	0.397	0.314	0.464
age 65–74	Medicare insurance coverage begins at age 65 for people who are eligible for Social Security benefits.		0.219	0.425	0.250	0.433
age 75+	Screening is not needed or recommended as often for older individuals who have had regular screening at younger ages.		0.189	0.463	0.325	0.468
**County-level predictors**						
managed care penetration (%)	Managed care has transformed the way medicine is practiced in highly-penetrated markets, with higher expected utilization of preventive care services (2005).	RTI Spatial Database (https://rtispatialdata.rti.org) is publicly/freely available. The database includes several geographic scales with data and boundary files for mapping.	15.9	14.7	15.3	14.7
Distance (miles)	Calculated as the average distance (miles) to provider based on all FFS Medicare residents in the county who utilized BC or CRC screening. Greater distance to provider of BC (mammogram) or CRC (endoscopy) screening suggests impeded access to preventive care services. Based on 100% FFS Medicare utilization of mammography or endoscopy services (2006).		6.02	6.10	5.15	4.80
Screening rate (%)	Percent of the 100% FFS Medicare population residing in the county and alive all year that utilized cancer screening (mammography, endoscopy) (2006).		23.60	3.18	11.05	1.43
Percent uninsured (%)	% of the under-age-65 population who did not have health insurance (2005).		17.73	5.45	17.75	5.49
**State-level Policy Variable**						
Direct Access to Specialist(1 = yes, 0 = no) in 2004See Figure 2 for each specific state's regulatory status	Access to gastroenterologists, gynecologists or oncologists without need of referral from a primary care physician may result in better matching of patient/provider and more timely care. Hypothesized to increase access for less well insured individuals or those in more stringent managed care plans. Younger people tend to be enrolled in these more stringent managed care plans, which are less costly but restrict access and choice. Source: NCSL, 2010.		0.956	0.206	0.951	0.216

### 2.3. Measures and Empirical Model

Outcome variable. Using the SEER summary stage 2000 variable provided in the database, we coded regional or distant diagnosis as late stage, and *in situ* or localized diagnosis as early stage. We then created a binary indicator for each individual specifying whether their cancer was diagnosed at a late stage, or not. In Table 1 (above) the overall proportion of BC cases diagnosed late is 0.308, while this is higher for CRC cases, at 0.543.

Person-level variables. We included four age groups, three race or ethnicity groups, and sex as compositional variables reflecting demographic characteristics of the cancer population. About 10–11% of the cancer population are black, and about 12% are other races combined; the vast majority are white, the reference group. Almost half (48.7%) of CRC cases are females. The omitted (reference) age group is age 50-64, which is the age span at which cancer screening is most strongly indicated for persons of average risk. In Table 1 (above) this group has the highest incidence of BC, while for CRC, the incidence rises again at the oldest age group. Generally speaking, BC incidence is higher among younger age groups than is CRC incidence. In the US during this time frame, BC screening protocols recommended screening to begin at age 40 for women of higher risk, while CRC screening guidelines recommended screening to begin at age 50 for persons of average risk, with no caveats for higher-risk groups.

County-level contextual variables. County of residence at time of diagnosis was used to match individuals to the county contextual measures. For measuring accessibility to screening services, we include an average distance to screening provider in the county, based on 100% FFS Medicare service utilization flows, available in a large public use database [28]. In Table 1 the average is about 5 miles for endoscopy and about 6 miles for mammography. Other variables from that database were included to reflect propensity to utilize breast or colorectal/endoscopic cancer screening among the county population. The screening variables are based on the 100% FFS Medicare BC or endoscopic CRC screening rates in the county of residence, which is the most broadly-based small-area measure available at the county level. For BC screening, about 24% of FFS Medicare-insured women utilized mammography in 2006. For CRC screening by endoscopy, about 11% of FFS Medicare-insured men and women utilized the service in 2006. We included managed care penetration to capture spillovers on area practice styles, following the recent literature which suggests there were significant managed care spillover effects on the utilization of endoscopy in 1999 [30] and its geographic availability during 2001–2005 [31]. Managed care penetration also reflects urbanicity. On average, about 15–16% of the population is insured by a managed care plan, but the standard deviation is large (almost 15%), reflecting the considerable heterogeneity in this measure across the landscape. We included the percent of the under-age-65 population who are uninsured as an additional access measure, which varies across states and is one of the strongest known predictors of late-stage cancer diagnosis [5]. The uninsured rate was about 18% in 2005, and it varied a good bit across the landscape (standard deviation about 5.5%). During this time frame, populations younger than age 65 were generally not as well insured as older individuals in the US, so it is important to control separately for age and uninsured rate, to disentangle these two effects.

State Regulations of Health Markets. In the empirical work which follows, we focus on one insurance regulation - the ‘Direct Access to Specialist’ mandate - that we hypothesize should be important in predicting cancer screening and incidence of late-stage diagnosis. This mandate requires insurance plans to cover direct access to specialists, without referrals from plan gatekeepers or primary care physicians, which could plausibly affect the availability of information regarding the importance of cancer screening. It may also impact the accessibility of specialists or physicians of choice to better align patients with the best medical advice. Ward et al. [5] found that people covered by the most preferred types of insurance (plans with the greatest freedom of choice of providers) were less likely to be diagnosed with late-stage cancer, while uninsured and Medicaid (public insurance for the poor and disabled) groups were more likely diagnosed at late stage. Thus timely access to preferred physicians and specialists seems important, and state regulations that enhance this may improve access.

**Figure 2.**
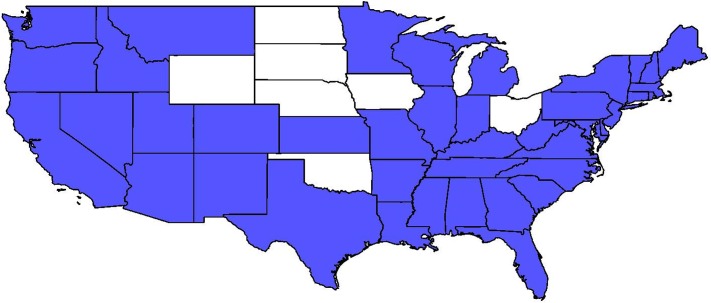
States Mandating Insurers to Cover Specialists Chosen Without Referral by Plan Providers, 2004 Legend: Blue = mandate in force; White = no such mandate.

The regulatory variable ‘Direct Access to Specialist’ is expected to enhance early-stage diagnosis outcomes, thus be negatively associated with late-stage diagnoses. Areas with higher proportions of uninsured individuals would realize less benefit from this regulatory mandate, as not having insurance would reduce the impact of the insurance regulation. We include a cross-level interaction between this state regulation and the county uninsured rate to assess whether the hypothesis that higher prevalence of uninsured would reduce the impact of the mandate. Figure 2 depicts the states with and without this mandate.

### 2.4. Statistical Analysis

We used multilevel models to examine associations with late stage cancer diagnosis from predictors at person, county and state levels. We specify a three-level random intercept logit model for the late-stage diagnosis with patients nested in counties which are nested in states. We used a multilevel modeling framework because we wanted to fit the regression to individuals while accounting for systematic, unexplained variation among counties and states. Ignoring the county and state level effects, when they are important, is tantamount to having omitted variables in the model, which can bias individual-level coefficient estimates. In addition, when the higher-level (e.g. county) covariates are of interest, failing to account for their structural similarity across individuals within the counties can yield biased standard errors for these county covariates, increasing their apparent statistical significance [11].

We modeled late-stage BC diagnoses separately from late-stage CRC diagnoses. We merged county-level community characteristics and the state-level regulatory variable with person-level records from the USCS registry data system. The three-level model, with the cross-level interaction term (area uninsured rate and state policy) was estimated using STATA GLLAMM (http://www.gllamm.org/). The models estimated included random intercept terms for county and state levels. Using these terms along with other estimated parameters, it is possible to predict the late-stage response for each individual, and then to aggregate these to the county level. The random intercept terms help ‘smooth’ the variability in the county predictions due to small sample sizes, increasing their robustness [11].

We use the multilevel modeling results to examine *Why* there are geographic disparities in late-stage cancer diagnoses (Table 2). We model the person's likelihood of late-stage BC diagnosis separately from the person's likelihood of late-stage CRC diagnoses. The BC model does not include males. For each cancer type, we provide estimates from a base model and from a model with cross-level interaction between county uninsured and state policy. The cross-level interaction between county uninsured and state policy is achieved by including these two variables separately in the model, and then multiplied together as a third term. We present model results for each cancer type with, then without this interaction term.

To answer questions related to *Where* disparities are observed, after estimation, model results were used to generate predicted county-level rates (proportions) of late stage diagnoses among all new cancers diagnoses during 2004–2009 for each type (BC, CRC). We generate the county level robust predictions of late-stage cancer diagnosis rates from the three-level, random-intercepts model, for CRC and BC cases in 40 of the 50 United States. We generate model-predicted late-stage cancer rates using the fitted values for the county and state random intercept terms along with other model estimates. The multilevel model used here accounts for ‘compositional’ characteristics of people in counties to yield estimates of county-level rates of late stage BC or CRC diagnosis that are adjusted for sample selection as well as sample size. The predicted rates of late-stage diagnosis from the model are thus smoothed relative to the distribution of raw rates. The county-level aggregate rates are defined as the average of predicted late-stage diagnosis among all cases in each county from each cancer model. County rankings are assessed relative to all counties included in the analysis of each cancer type. The county-level rates are ranked and then grouped into the lower (Q1), middle (M), and upper (Q4) quantiles across all counties in the 40 states. The confidence intervals for the means of the upper and lower quantiles are quite narrow, and allow us to conclude that counties ranked in the upper quantile of the late-stage distribution are significantly different from counties ranked in the lower quantile of the rates distribution.

**Figure 3:**
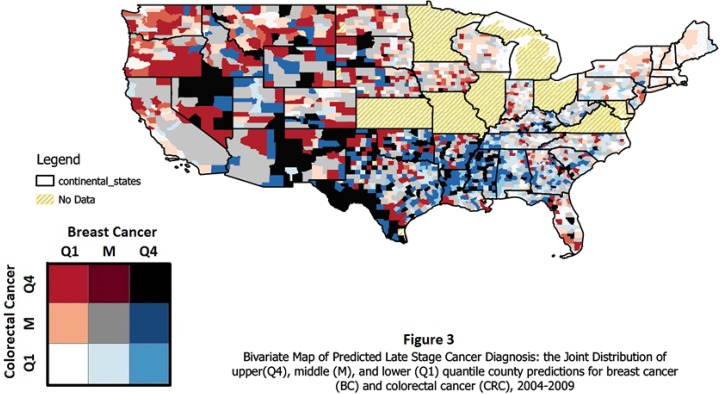
Bivariate Map of Predicted Late Stage Cancer Diagnosis: the Joint Distribution of upper (Q4), middle (M), and lower (Q1) quantile country predictions for breast cancer (BC) and colorectal cancer (CRC), 2004-2009

To spatially translate the findings from the modeling, we combined the county ranking for CRC and BC together in one map after calculating the different combinations in the bivariate (joint) distribution of the BC and CRC rankings. There are nine possible combinations, and each is color coded with a different color. We use the Color Brewer palette (colorbrewer2.org) to choose a color scheme that is legible to color blind individuals. The bivariate legend allows the viewer to assess whether one or both cancers have higher quantile rates in a particular state. For example, in Figure 3, Q1 along the colorectal cancer axis in the grid legend designates counties in the lowest quantile of the predicted late-stage CRC rates while Q4 designates counties in the highest quantile of the predicted late-stage CRC rates, and M designates counties that are between the upper and lower quantiles, in the middle of the distribution. Similarly, Q1, M, and Q4 along the breast cancer axis in the legend are designated. The palette chosen for the bivariate legend has lighter colors for areas where counties are in the lower or middle quantiles for both cancer types (white and grey, see Figure 3), or middle for one and low for the other (pink and light blue). The more vivid and darker the shade, the higher the quantile. Red and dark red are upper quantile for CRC, while blue and dark blue are upper quantile for BC. The black square in the legend represents counties where both BC and CRC diagnosis rates are in the upper quantile. The results are displayed in Figure 3, discussed in the next section.

## 3. Results

### 3.1. Multilevel Regression Results

Table 2 presents the regression results. Age is an important predictor for both cancer types. Younger people are more likely than the age 50-64 reference group to be diagnosed with late-stage cancer. This association is much larger for the CRC than the BC cases (0.306 versus 0.040) while both are highly significant. This most likely reflects the fact that the cancer screening guidelines for BC had been adapted to include higher-risk individuals, who were encouraged to utilize screening at younger ages, while no such adaptation had emerged in the CRC screening guidelines. For people over age 65, the likelihood of late-stage diagnosis is lower than for people in the reference group, which was defined to coincide with prime screening age for average-risk persons. The decline with age is significant for both cancer types, but much more pronounced (larger effect size) for BC than for CRC. Females and blacks are significantly more likely to be diagnosed at late stage for CRC than males and whites. For BC, both blacks and other minorities are more likely to be diagnosed late than whites, and these disparities are quite large—larger than the black-white disparity noted for CRC. All of the person-level predictors are highly statistically significant in both cancer type models.

Holding constant statistically the compositional (person-level) factors, we can assess the associations between late-stage diagnosis and contextual factors unbiased by selection effects [32]. Managed care penetration is the largest contextual effect, and it has opposite signs in the BC and CRC models. In BC models, living in a county with higher managed care penetration is associated with statistically significant and lower likelihood of late-stage BC diagnosis. In CRC models, living in a county with higher managed care penetration is associated with statistically significant and higher likelihood of late-stage BC diagnosis. Area BC and CRC screening rates are the predictors with the next largest effect size. Living in counties where higher percentages of FFS Medicare elderly undergo BC and CRC screening is associated with lower likelihood of late-stage diagnosis of either cancer. Distance to provider of screening services is significant for the CRC model, but not for the BC model. Finally, the percentage of population without health insurance is a statistically significant, albeit small positive effect in both models.

In the models without the interaction term, the state policy variable ‘Direct Access to Specialist’ is statistically significant and negative. This suggests that individuals living in states that have enacted this mandate have lower likelihood of late-stage diagnosis than individuals living in states that have not enacted it, after accounting statistically for everything else in the model. The effect size is greater than the county contextual factor screening, but smaller than the county contextual factor managed care penetration. When interacted with county uninsured in the CRC model, the estimate is statistically significant and we can conclude that this mandate is less effective in areas with higher uninsured rates. The interaction term is not significant in the BC model.

**Table 2.** Multilevel Modeling Results for BC and CRC: Predictors of Late-Stage Diagnosis.

	CRC		CRC (interaction)		BC		BC(interaction)	
	coeff	pval	coeff	pval	coeff	pval	coeff	pval
**Person level**								
Age < 50 (reference 50–64)	0.306	0.000	0.306	0.000	0.040	0.000	0.040	0.000
Age 65-74	−0.105	0.000	−0.105	0.000	−0.255	0.000	−0.255	0.000
Age 75+	−0.051	0.000	−0.051	0.000	−0.139	0.000	−0.139	0.000
Female (reference male)	0.040	0.000	0.040	0.000	.	.	.	.
Black (reference white)	0.079	0.000	0.079	0.000	0.386	0.000	0.386	0.000
Race all other (reference white)	0.008	0.363	0.008	0.399	0.136	0.000	0.136	0.000
**County level**								
Percentage of population < age 65, with no health insurance	0.005	0.000	0.007	0.016	0.003	0.002	0.005	0.031
Average distance traveled by Medicare beneficiary to cancer screening provider	0.002	0.037	0.002	0.041	0.000	0.532	0.000	0.519
Screening rate (percent of area FFS Medicare population utilizing screening)	−0.036	0.000	−0.037	0.000	−0.027	0.000	−0.027	0.000
Managed care penetration (percentage of area insured population in managed care plans)	0.101	0.033	0.103	0.039	−0.284	0.000	−0.285	0.000
**State level**								
State Policy (1 = yes, 0 = no) ‘Direct Access Specialist’	−0.055	0.004	−0.271	0.000	−0.053	0.001	−0.009	0.841
**Cross level interaction**								
< 65 Pop uninsured* state policy interaction	.	.	0.013	0.000	.	.	−0.003	0.288
**Variance Components**								
Level 1 * (individual)	3.2899		3.2899		3.2899		3.2899	
Level 2 (county)	0.02497		0.02484		0.01450		0.01447	
Level 3 (state)	0.00074		0.00065		0.00018		0.00017	

*For logistic multilevel models, the variance for level one is assumed to be π^2^/3.

To determine the importance of information available in the random intercept terms, it is customary to look at the variance components estimated as model parameters. The variance of the random intercepts at the county and state levels are very small (bottom rows Table 2). Two fairly recent papers examining late-stage BC diagnosis in California used the random intercepts multilevel model and found similarly small variance components [2][33]. We conclude, as they do, that a small variance estimate for the area-level random effects indicates that the contextual factors included in the model do a good job accounting for spatial heterogeneity in the explanatory factors.

### 3.2. Spatial Translation to Policy Map

The non-interacted models in Table 2 are used for mapping to translate the findings. Taken as a group, the person-level factors are the strongest predictors in these models. Among the contextual factors, the managed care penetration and state policy climate are the most important predictors. The model fitted on the covariates and random intercepts produces the predictions used in the translational mapping. Figure 3 delivers the translational mapping, which depicts the bivariate distribution of the counties ranked according to the distribution of values for county late-stage rates defines for all counties in the 40 states, mapped in Figure 3. At a glance, one can see that the distribution of rankings across the US is not uniform, and that some states have much more challenging work ahead of them in cancer control than do others.

Some states in the Northeast compare favorably with the rest of the country, as their coloring indicates pale shades of pink and blue, grey and white. Some states exhibit only a few counties with either red, blue, or black coloring which indicate those counties as problem areas that may deserve more focused attention. In the South Central portion of the map, there is lots of blue, indicating high rates of late-stage BC clustered geographically in the region. Counties in the states in the West are larger, so the viewer should not be fooled by the large black patches that represent a few counties with high quantile rates for both cancers. The state of California on the West coast is doing relatively well, but problems with high rates of late-stage CRC are evident in the San Francisco Bay area, the adjacent North coast, and inland from the Bay area.

## 4. Discussion

The regression findings are the predictions on which the maps are based. Some covariates were more important than others in terms of magnitudes of effect that would come through in the predicted patterns. The main drivers of the patterns seen here are the demographic variables, which are consistently strong predictors in the models. Disparities related to age are strong predictors, especially in the CRC model. The regression results suggested that minority disparities are greater for BC than for CRC, as the effect sizes on the minority coefficients are larger - thus it is not surprising that we see a cluster of high-quantile blue areas in the South Central US, along the Mississippi River where prevalence of poor black communities is higher. Similarly, the dark areas along the Western coast of Texas may represent poor Hispanics concentrated in the Rio Grande area. Other dark patches may represent poor Native American communities concentrated in the four corners area (of Utah, Colorado, Arizona, and New Mexico), Oklahoma, and South Dakota.

Holding constant statistically the effects of these compositional (demographic) variables describing the study population, we can assess associations between county-level contextual predictors and outcomes that are free from selection bias. This aspect of multilevel modeling was stressed by Oakes [32] and is important here where several of the contextual variables vary considerably in their prevalence across the landscape. Managed care prevalence is the strongest county area-level contextual predictor. Managed care prevalence is higher in urban areas, and has a strong negative association with the likelihood of late-stage BC diagnosis. California has the highest managed care penetration of any state in the nation, and was a historical leader in this type of insurance [34]. Thus it is not surprising that the map shows few late-stage BC areas of concern in California. While managed care practices have been promoting mammography screening for several decades, consistent with the large, statistically significant negative effect in the BC model—the CRC screening guidelines were evolving during this period and managed care plans were slow to migrate away from cheaper screening modalities to more costly endoscopy without a strong cost-effectiveness basis [31],[35]. Thus, consistent with the notion that managed care plans did not strongly endorse endoscopy during this period, evidence suggests that living in a county with higher managed care penetration is associated with a higher likelihood of late-stage CRC diagnosis.

Area BC and CRC screening rates are the predictors with the next largest effect size. Living in counties where higher percentages of FFS Medicare elderly undergo BC and CRC screening is associated with lower likelihood of late-stage diagnosis of either cancer. This suggests that Medicare screening rates are good proxies for population screening behaviors more generally. While data do not permit knowledge of personal screening history, it is apparent that area-level contextual factors are important predictors of individual behaviors. Distance to provider of screening services is significant for the CRC model, but not for the BC model. This difference most likely reflects the fact that CRC screening by endoscopy requires help from another person getting to and from the appointment, while mammography does not. Finally, the percentage of population without health insurance is a statistically significant, albeit small positive effect (increasing likelihood of late-stage diagnosis) in both models. Lack of health insurance in the area likely characterizes a population that has lower demand for healthcare services, and is less inclined to think about their health or utilize preventive care services. Such areas are also unattractive to providers of screening services who must invest in infrastructure to serve the local demand [31].

The state-level regulatory variable ‘Direct Access to Specialist’ was included in the model as the highest level construct. This mandate requires insurance plans to cover direct access to specialists, without referrals from plan gatekeepers or primary care physicians, which could plausibly affect the availability of information regarding the importance of cancer screening. It may also impact the accessibility of specialists or physicians of choice to better align patients with the best medical advice. Ward et al. [5] found that people covered by the most preferred types of insurance (plans with the greatest freedom of choice of providers) were less likely to be diagnosed with late-stage cancer, while uninsured and Medicaid (public insurance for the poor and disabled) groups were more likely diagnosed at late stage. Thus timely access to preferred physicians and specialists seems important, and state regulations that enhance this may improve access.

We hypothesized that this mandate would be important in predicting cancer screening and incidence of late-stage diagnosis. Thus, we expected this covariate to enhance early-stage diagnosis outcomes, thus be negatively associated with late-stage diagnoses. We also hypothesized that areas with higher proportions of uninsured individuals would realize less benefit from this regulatory mandate, as not having insurance would reduce the impact of the insurance regulation. We included a cross-level interaction between this state regulation and the county uninsured rate to assess whether the hypothesis that higher prevalence of uninsured would reduce the impact of the mandate. We find that the state policy variable ‘Direct Access to Specialist’ is statistically significant and negative. This suggests that individuals living in states that have enacted this mandate have lower likelihood of late-stage diagnosis than individuals living in states that have not enacted it. When interacted with county uninsured in the CRC model, we can conclude that this mandate is less effective in areas with higher uninsured rates. This is what we would expect, because uninsured people would not be impacted by such a mandate. The interaction term is not significant in the BC model.

## 5. Conclusion

Differences in estimation results between the BC and CRC model suggest that the cancer control trajectories for these two cancer types are different. To prevent late-stage diagnoses, cancer screening must be effective at catching cancer early or in preventing development of cancer. While mammography has been accepted as the optimal BC screening method for women of average risk for decades, it was not until about 2008 that colonoscopy was recognized as an important prevention and early detection tool in the official CRC screening guidelines [35]. During this period, insurers typically covered BC screening entirely, but have been more reluctant to cover endoscopic screening for CRC and have imposed sizeable out-of-pocket costs to consumers when coverage was provided. Thus, it is not surprising that BC screening rates have been higher than CRC screening rates (Table 1) and subsequently, rates of late-stage BC are lower than rates of late-stage CRC overall. However, disparities between minorities and whites are greater for late-stage diagnoses of BC than CRC. This suggests that the minorities are harder to reach populations needing continued focus from comprehensive breast cancer control efforts. For CRC, age < 50 is the greatest risk factor for late-stage diagnosis, with much larger effect size than race or ethnicity. This suggests that the younger than age 50 population may need targeted efforts to reduce the incidence of late-stage diagnosis.

There are also geographic differences in the rates of late-stage diagnosis for these two cancer types. Some areas have high rates of late-stage BC, while others have high rates of late-stage CRC, and some areas have high rates of both. Translational mapping is an effective tool that can be used to translate how one state and its counties compare to others, which may help states to assess whether their current cancer control efforts are on target. In this paper, counties where the predicted rates of late-stage outcomes are in the upper and lower quadrants of the distribution across all counties are identified on a map, demonstrating these spatial patterns. Using maps showing the bivariate distribution of these predictions across both BC and CRC cancer types, geographic disparities are revealed. The results compare counties in individual states to counties across all 40 states examined, providing a relative comparison that enables assessment of how one state's cancer control efforts might compare with others. Each state in the US has decentralized funding and control of such efforts, so such multistate assessments are rare. We welcome contact with state cancer control or central cancer registry officials who may want to discuss these results, or learn how to conduct further explorations of their own inside the secure RDC environment to inform local policies.

Multilevel modeling using a random intercepts formulation is a valuable method for producing robust, area-level rates of disease for public health research, when population data are available. This approach is based on the population data and does not interpolate values for areas without any data. The translational mapping of the multilevel modeling predictions is a viable tool to assist in identifying geographic disparities and sharpening the focus on areas with higher disease rates or rates of poor outcomes, such as late-stage cancer diagnosis.

## List of Abbreviations Used in the Paper

BC: breast cancer
CDC: Centers for Disease Control and Prevention
CRC: colorectal cancer
NCHS: National Center for Health Statistics
NCI: National Cancer Institute
NCSL: National Conference of State Legislatures
RDC: Research Data Center
SEER: surveillance, epidemiology, and end results
USCS: United States Cancer Statistics
